# Morpho-physiological and proteomic responses to water stress in two contrasting tobacco varieties

**DOI:** 10.1038/s41598-019-54995-1

**Published:** 2019-12-06

**Authors:** Zheng Chen, Jiayang Xu, Fazhan Wang, Lin Wang, Zicheng Xu

**Affiliations:** 1 College of Tobacco Science, Henan Agricultural University, Zhengzhou, 450002 China; 2 Agronomy and Biotechnology College, China Agricultural University, Beijing, 100193 China

**Keywords:** Plant physiology, Plant molecular biology, Plant ecology

## Abstract

To gain insight into the molecular mechanisms underpinning tobacco (*Nicotiana tabacum*) tolerance to drought stress, we integrated anatomical, physiological, and proteomic analyses of drought-tolerant (Yuyan6, [Y6]) and -sensitive (Yunyan87 [Y87]) varieties. In comparison to Y87, Y6 exhibited higher water retention capability, improved photosynthetic performance, delayed leaf-senescence, stable leaf ultrastructure, a stronger antioxidant defense, and lesser ROS accumulation when subjected to water stress. Using an iTRAQ-based proteomics approach, 405 and 1,560 differentially accumulated proteins (DAPs) were identified from Y6 and Y87 plants, respectively, of which 114 were found to be present in both cultivars. A subsequent functional characterization analysis revealed that these DAPs were significantly enriched in eight biological processes, six molecular functions, and six cellular components and displayed differential expression patterns in Y6 and Y87 plants, suggesting that the response to water stress between both varieties differed at the proteomic level. Furthermore, we constructed protein coexpression networks and identified hub proteins regulating tobacco defenses to water stress. Additionally, qPCR analysis indicated that the majority of genes encoding selected proteins showed consistency between mRNA levels and their corresponding protein expression levels. Our results provide new insights into the genetic regulatory mechanisms associated with drought response in tobacco plants.

## Introduction

Among abiotic stresses, drought is of particular concern given that it can strongly affect plant survival and productivity, especially in arid environments^1,2^. Drought events have been predicted to increase steadily with ongoing global warming scenarios due to climate change^3^. Understanding the mechanisms that regulate plant growth during drought conditions is currently one of the central issues of plant biology research. Water deprivation can trigger a suite of modifications at the molecular, cellular, and physiological levels, hindering plant growth^4–6^. Previous studies have proposed that water limitation can reduce biomass accumulation, disrupt cellular homeostasis, damage chloroplast structure, constrain photosynthesis, facilitate the production of reactive oxygen species (ROS), and increase lipid peroxidation, ultimately leading to death^7–11^. Additionally, excess light energy induced by drought decreases photosynthetic activity, leading to photoinhibition and even photooxidation in stressed plants^12^. To cope with unfavorable environmental conditions, plants have evolved sophisticated defense strategies at multiple levels to maintain their growth, development, and cellular processes. During plant evolution, the efficient action of a complex adaptive mechanism, which involves stress signal transduction networks, elevated abscisic acid (ABA) levels, stomatal regulation, increased accumulation of antioxidants and osmoprotectants, and stress-responsive gene expression, improved plant resistance to drought^7,13–18^. Even though drought tolerance has proven difficult to define, as it is a multigenic trait that involves a large number of genes^19^, a continuous effort to elucidate the molecular basis of drought tolerance is required in order to cultivate crops with enhanced water-use efficiency and to maintain environmental sustainability. This is especially true when considering the elevated drought severity caused by climate anomalies and the uncertainty over future water supplies for an increasing global population.

Tobacco (*Nicotiana tabacum*) is a model plant for genomic research and an economically influential crop in China. Compared to other crop species, it is relatively more sensitive to insufficient water supplies. Previous studies with tobacco plants have primarily focused on abiotic stress tolerance, disease resistance, secondary metabolites, and transcriptomic expression, especially in transgenic varieties^20–29^. However, information regarding different drought response strategies in non-transgenic tobacco cultivars is scarce. In recent years, many high-throughput approaches, such as transcriptomics, proteomics, and metabolomics, have been applied to identify differences in stress responses among divergent crop varieties^30–32^. Although several genes have been identified in different crop species by transcriptomic analysis, protein levels cannot be accurately estimated from mRNA levels in most situations^33,34^. Thus, using proteomic analysis of contrasting drought-tolerant tobacco varieties is necessary.

We have a longstanding interest in providing further insights into the response of Solanaceae crop species to insufficient water supplies. Here, we attempted to determine the proteomic differences between two different varieties of tobacco in response to short-term water stress. We performed an iTRAQ-based quantitative proteomics analysis to determine the responses of Yuyan6 (Y6) and Yunyan87 (Y87) cultivars to polyethylene glycol (PEG)-induced drought stress. These results will help identify drought-resistant proteins among the differentially abundant proteins (DAPs) and provide new approaches for further molecular breeding of drought-resistant tobacco plants.

## Results

### Analysis of drought tolerance of both tobacco varieties

**Figure 1:**
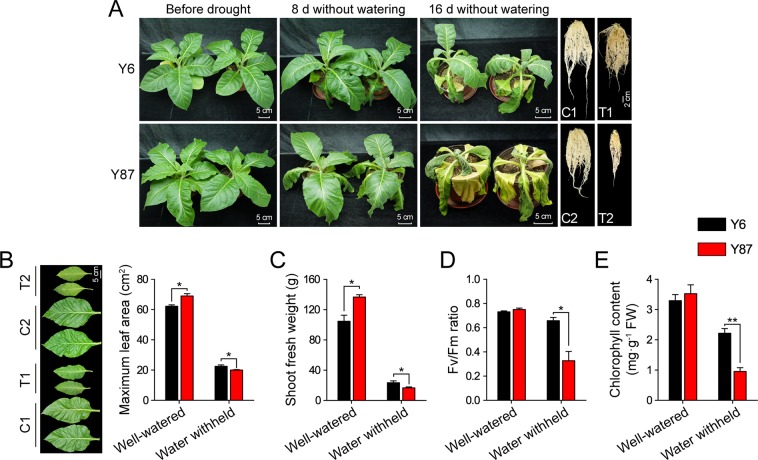
Drought tolerance of Y6 and Y87 varieties. (**A**) Growth performance after withholding water for 8 and 16 days. (**B**–**E**) Leaf area, fresh weight, Fv/Fm ratio, and chlorophyll content in Y6 and Y87 plants after withholding water for 16 days. Y6 and Y87 plants grown under well-watered conditions were designated as C1 and C2, respectively, and when exposed to drought conditions, were labeled as T1 and T2, respectively. Values represent mean ± SE of three replicated experiments. Asterisks indicate significant differences between Y6 and Y87 plants (Student’s *t*-test; *p < 0.05, **p < 0.01).

To test the drought tolerance of Y6 and Y87, 8-week-old soil-grown seedlings were exposed to progressive drought treatment. After 16 days of water deprivation, Y87 showed severe drooping and wilting, while Y6 exhibited more open, greener leaves and lower levels of root growth inhibition (Fig. 1A). When exposed to long-term drought conditions, Y87 showed reduced leaf area and fresh plant weight, decreased Fv/Fm ratio, and significant decreases in chlorophyll content in comparison to Y6 (Fig. 1B–E). These results clearly demonstrate that Y6 was considerably more tolerant to drought than Y87.

### Morphological changes in both varieties under water stress

**Figure 2:**
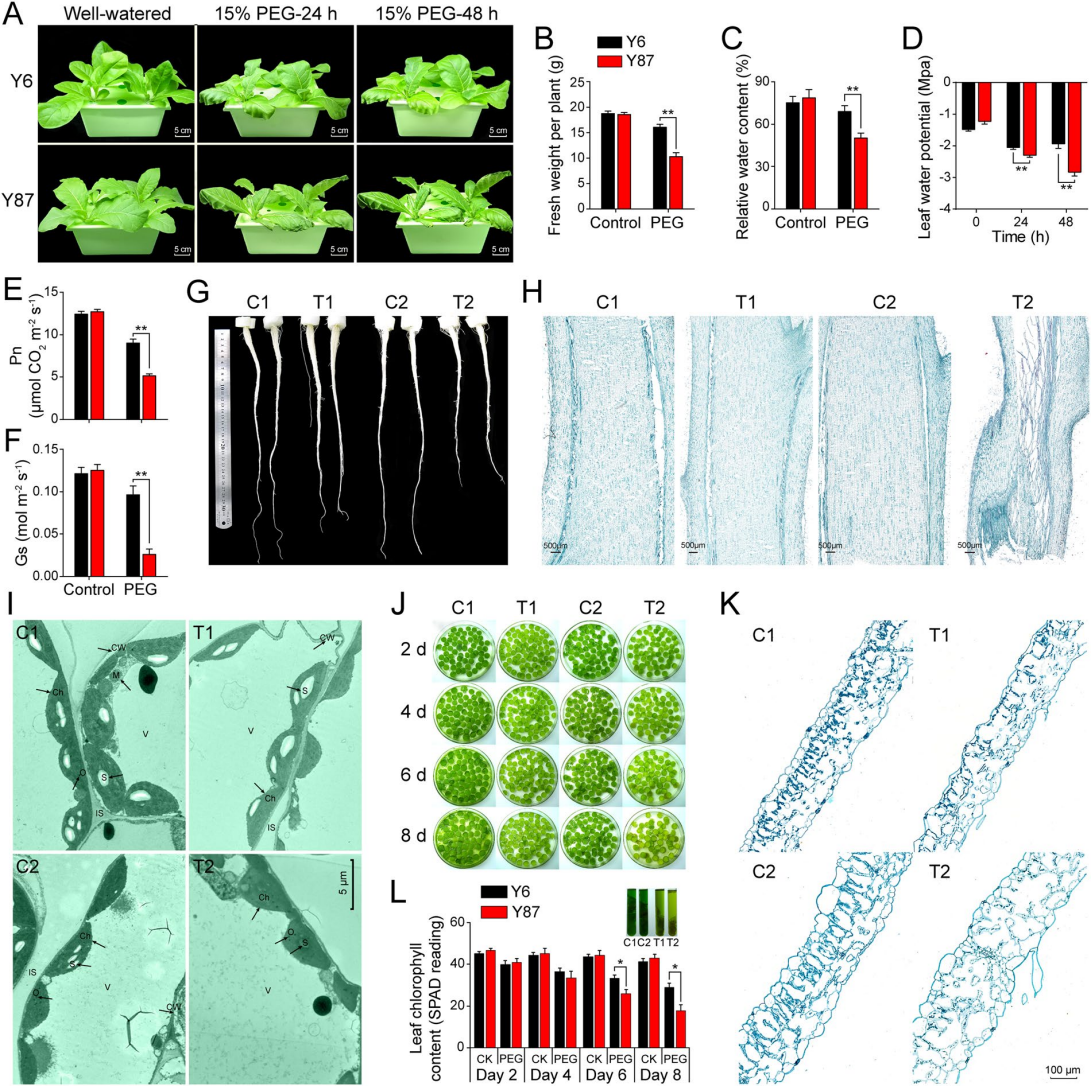
Morphological changes in seedling of Y6 and Y87 varieties after PEG-induced water stress. (**A**) Growth performance of Y6 and Y87 before and after the PEG treatment. (**B**–**D**) Fresh weight, relative water content (RWC), and leaf water potential under well-watered conditions (control) and after two days of PEG-induced water stress. (**E**,**F**) Photosynthetic rate and stomatal conductance under well-watered conditions (control) and after two days of PEG-induced water stress. (**G**) Root length of Y6 and Y87 seedlings treated with PEG for two days. (**H**) Longitudinal sections of the stems of Y6 and Y87 plants. (**I**) Mesophyll chloroplasts in normal and stressed leaves observed with transmission electron microscopy. Labels: Ch, chloroplast; S, starch grain; M, mitochondrion; V, vacuole; O, osmiophilic plastolobuli; CW, cell wall; IS, intercellular space. (**J**) Leaf disc assay of Y6 and Y87 plants during normal and with 15% PEG-induced water stress. (**K**) Transverse sections of leaf discs from Y6 and Y87 plants treated with 15% PEG for six days. (**L**) Chlorophyll loss in well-watered (CK) and PEG-stressed leaf discs. The inset graph above (L) represents the chlorophyll extraction solutions of leaf discs (n = 10) at the end of the stress period. Values represent mean ± SE of three replicated experiments. Asterisks indicate significant differences between Y6 and Y87 plants (Student’s *t*-test; *p < 0.05, **p < 0.01).

To investigate morphological changes in Y6 and Y87 after PEG-induced water stress, we initially evaluated the seed germination percentages of the two varieties. In the presence of 15% PEG, seeds of the Y6 variety showed greater germination percentages (91.7%) in comparison to Y87 (28%; Fig. S1). When 6-week-old seedlings were exposed to PEG-induced water stress for two days, leaves of Y87 seedlings showed serious wilting, while Y6 seedlings were affected to a lesser extent (Fig. 2A). Fresh weight and relative water content (RWC) of both varieties did not differ in well-watered conditions, but water deficit reduced fresh weight and RWC of Y87 plants in comparison to Y6 plants (Fig. 2B,C). As the time withholding water increased, leaf water potential decreased in both Y6 and Y87, but at a much higher rate in Y87 (46.4% in two days) than in Y6 (Fig. 2D). After 48 h of PEG-induced water stress, significantly lower photosynthetic rate (Pn) and stomatal conductance (Gs) were observed in Y87 leaves in comparison to Y6 seedlings (Fig. 2E,F). Root length of Y87 decreased by 38% in relation to Y6 when subjected to two days of water stress (Fig. 2G), indicating that Y6 plants were more capable of maintaining root elongation during drought conditions than Y87 plants. To compare the morphological difference between both varieties, we performed safranin O/fast green FCF staining. We observed significant morphological injury in the stems of Y87 plants in comparison to Y6 plants (Fig. 2H), indicating that drought conditions suppressed stem lignification in Y87 plants. Further observations using a transmission electron microscope (TEM) revealed that, under well-watered conditions, chloroplast ultrastructure was similar in both varieties. However, after PEG-induced water stress, ultrastructural alterations were considerably more pronounced in Y87 than in Y6 plants, where Y87 plants exhibited irregular chloroplast morphology and a reduced number of chloroplasts and starch granules in relation to Y6 (Fig. 2I, Fig. S2).

To analyze differences in dehydration-induced leaf senescence between Y6 and Y87, an *in vitro* leaf senescence assay was performed. With increasing time of the water stress treatment, the intensity of stress-induced leaf senescence also increased, particularly in leaf discs from Y87, indicating that Y87 plants exhibited leaf senescence earlier than Y6 (Fig. 2J). The anatomical structure of leaf discs treated with PEG for 8 days indicated that the mesophyll cell density was lower in Y87 plants in relation to Y6 plants (Fig. 2K), which resulted in Y6 leaves being noticeably greener. Leaf senescence is typically accompanied by a reduction in chlorophyll content, corroborated by the SPAD chlorophyll meter readings of leaf discs, which showed a 44% decrease in the chlorophyll content of Y87 plants on day 8, but only a 21% decrease in Y6 plants, when compared to their corresponding controls (Fig. 2L). These findings suggest that Y6 plants exhibited delayed leaf-senescence and improved water status compared to Y87 plants under water stress.

### Water loss and stomatal responses induced by water stress in both varieties

**Figure 3:**
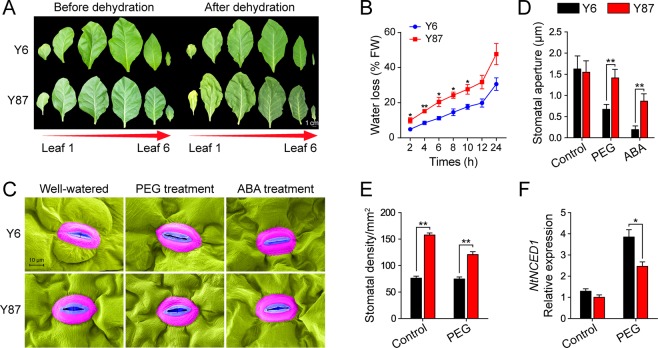
Analysis of water loss and stomatal status. (**A**) Phenotypic comparisons of detached leaves after dehydration for one day. (**B**) Kinetics of water loss from leaves (third leaf) of Y6 and Y87 plants during 24 h of dehydration. (**C**) Scanning electron microscope images of stomatal apertures of Y6 and Y87 plants treated with 15% PEG and 100 μM ABA solution. (**D**) Average width of stomatal apertures in Y6 and Y87 plants under well-watered conditions and treated with 15% PEG or 100 μM ABA solution (n = 15). (**E**) Stomatal density in Y6 and Y87 plants under well-watered conditions and treated with 15% PEG. (**F**) Relative expression level of *NtNCED1* in Y6 and Y87 plants under well-watered conditions and treated with 15% PEG. The expression level in Y87 plants under well-watered condition was defined as 1.0. Values represent mean ± SE of three replicated experiments. Asterisks indicate significant differences between Y6 and Y87 plants (Student’s *t*-test; *p < 0.05, **p < 0.01).

To further examine the water-retention differences between Y6 and Y87, we determined water loss in detached leaves. Compared to Y6 plants, leaves from Y87 plants showed severe coiling after 24 h of dehydration (Fig. 3A). Consistently, detached leaves of Y6 plants showed lower water loss rates than those of Y87 (Fig. 3B).

There were no differences in stomatal apertures between Y6 and Y87 under well-watered conditions. However, when analyzing leaf stomatal apertures in Y6 and Y87 plants exposed to PEG-induced water stress and to ABA, we observed significantly smaller stomatal apertures in Y6 leaves in comparison to Y87 leaves (Fig. 3C,D). Interestingly, Y6 exhibited lower stomatal density than Y87 under both normal and PEG-induced water stress conditions, indicating that Y87 plants were more likely to lose water in response to water stress (Fig. 3E, Fig. S3). We further examined the levels of ABA-related gene transcripts using qPCR, which revealed that, after PEG-induced water stress, Y6 plants had higher levels of *NtNCED1* expression in relation to Y87 plants (Fig. 3F).

### Changes in oxidative damages and antioxidant defenses of both varieties under water stress

**Figure 4:**
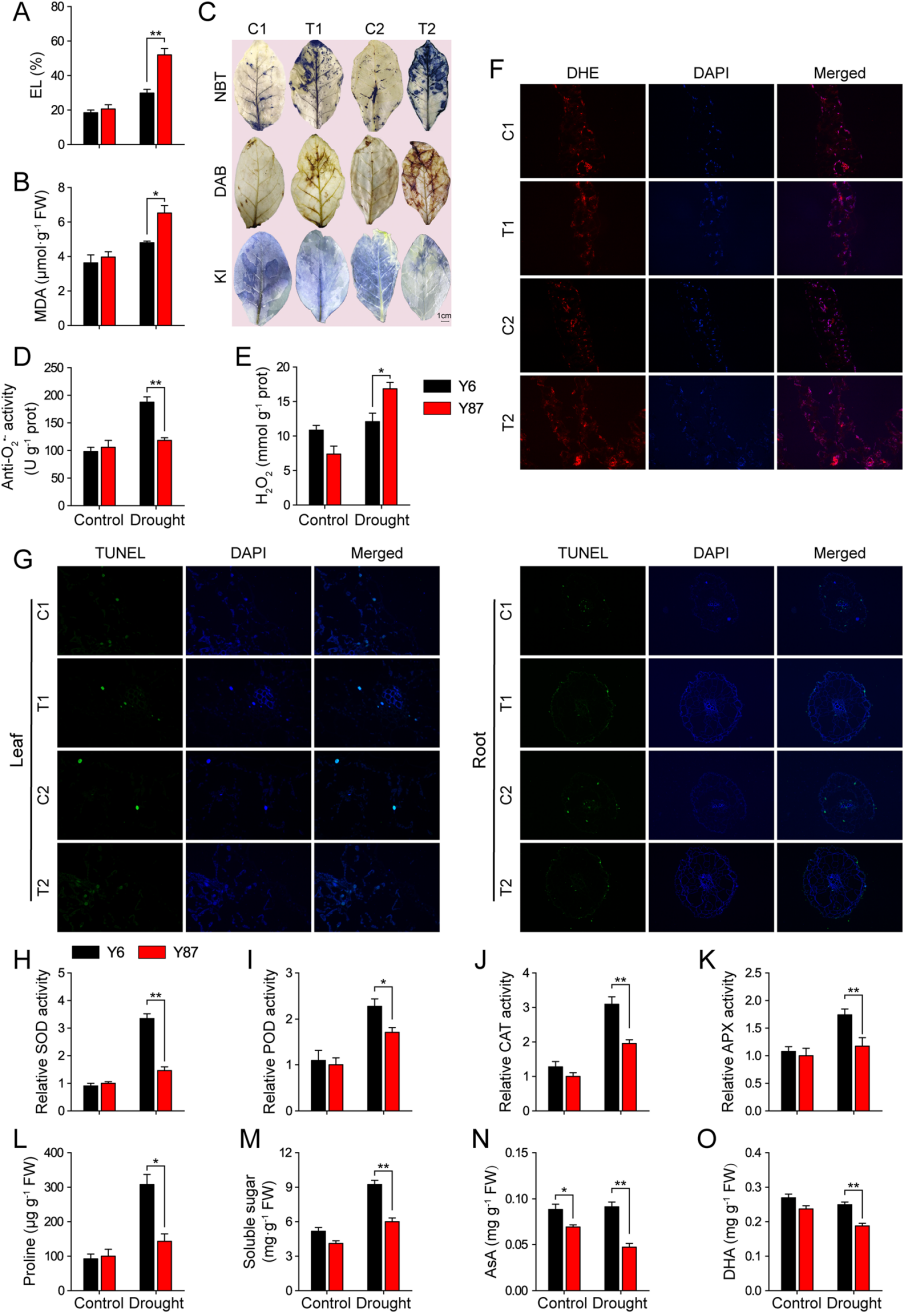
Biochemical analyses of Y6 and Y87 plants under well-watered conditions (control) and PEG-induced water stress. (**A**) EL and (**B**) MDA content. (**C**) *In vivo* localization of O_2_^•−^, H_2_O_2_, and starch by nitroblue tetrazolium (NBT), diaminobenzidine (DAB), and potassium iodide (KI) staining, respectively. (**D**,**E**) Determination of O_2_^•−^ production rate and H_2_O_2_ levels. (**F**) Fluorescence microscopic analysis of reactive oxygen species (ROS) accumulation with dihydroethidium (DHE) and 4,6-diamidino-2-phenylindole (DAPI) staining; merged images are also shown. (**G**) TUNEL assay of the programmed cell death process after each treatment with TUNEL and DAPI staining; merged images are also shown. (**H**–**K**) Antioxidant enzyme activities; relative activities of superoxide dismutase (SOD), peroxidase (POD), catalase (CAT), and ascorbate peroxidase (APX) in Y87 under normal condition were defined as 1.0. (**L**–**O**) Non-enzymatic antioxidants (proline, soluble sugars, ascorbic acid [AsA], and dehydroascorbate [DHA]). Values represent mean ± SE of three replicated experiments. Asterisks indicate significant differences between Y6 and Y87 plants (Student’s *t*-test; *p < 0.05, **p < 0.01).

Under PEG-induced water stress, Y6 plants displayed lower electrolyte leakage (EL) and a concomitant decrease in malondialdehyde (MDA) content in comparison to Y87 plants, indicating that Y6 plants experienced less cell membrane damage than Y87 plants (Fig. 4A,B). Histochemical staining showed that ROS levels in both seedlings were higher in relation to their corresponding controls, but after PEG-induced water stress, more intense staining was observed in Y87 leaves than in Y6 leaves. Conversely, under water stress, starch accumulation was less in Y87 plants than in Y6 plants (Fig. 4C). Further quantitative analyses indicated that the levels of O_2_^•−^ and H_2_O_2_ were markedly higher in Y87 leaves than in Y6 leaves after PEG-induced water stress (Fig. 4D,E). Consistent with the quantitative assay results, fluorescence microscopic analysis of ROS accumulation also showed that Y6 plants accumulated less ROS than Y87 plants under water stress (Fig. 4F). The TUNEL assays of programmed cell death (PCD) revealed that, under PEG-induced water stress, the number of TUNEL-positive cells was markedly higher in leaves and roots of Y87 plants than in leaves and roots of Y6 plants (Fig. 4G, Fig. S4). To compare the differences of the antioxidant defense systems between Y6 and Y87, we examined ROS-scavenging activity. Under normal conditions, the activity of superoxide dismutase (SOD), peroxidase (POD), catalase (CAT), and ascorbate peroxidase (APX) were not significantly different between Y6 and Y87. However, after PEG-induced water stress, the levels of these antioxidant enzymes were higher in Y6 plants than in Y87 plants (Fig. 4H–K). Similarly, the accumulation of osmolytes (proline and soluble sugars) and non-enzymatic antioxidants (ascorbic acid [AsA] and dehydroascorbate [DHA]) was significantly higher in Y6 plants in comparison to Y87 plants under PEG-induced water stress (Fig. 4L–O). These results suggest that the higher oxidative stress resistance of Y6 plants under water stress, resulted from ROS homeostasis as they showed enhanced antioxidant potential.

### Proteomic changes in both varieties under water stress

**Figure 5:**
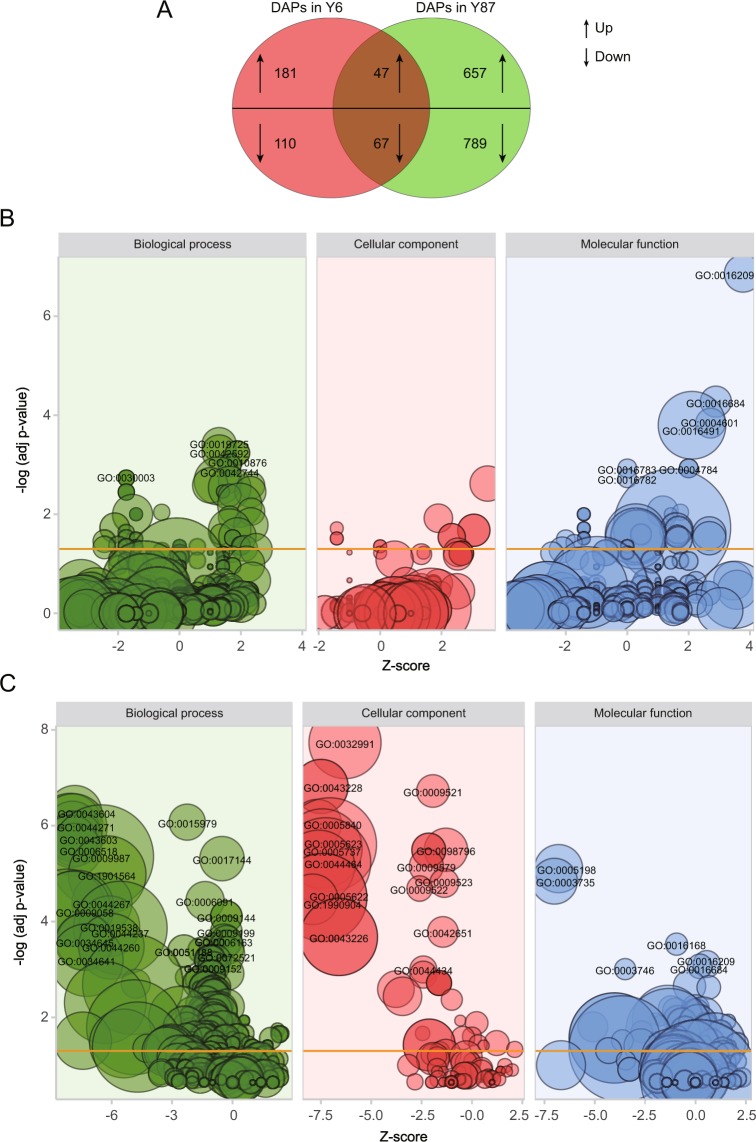
Graphical representation and gene ontology analysis of differentially accumulated proteins (DAPs) in both varieties. (**A**) Venn diagram analysis of the number of DAPs in Y6 and Y87. (**B**) Gene ontology annotations for DAPs in Y6. (**C**) Gene ontology annotations for DAPs in Y87. The area of the displayed circles is proportional to the number of proteins assigned to the GO term. The z-score is assigned to the x-axis and the negative logarithm of the adjusted p-value to the y-axis.

**Figure 6:**
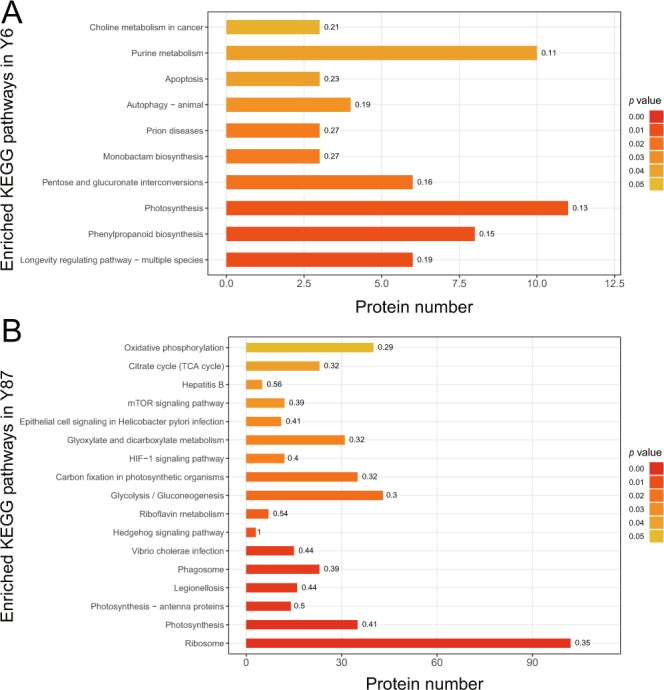
KEGG pathway analysis of DAPs in Y6 (**A**) and Y87 (**B**) varieties after water stress.

To characterize the proteomic responses of Y6 and Y87 to water stress, a comparative proteomics analysis was performed. We successfully identified and quantified 6,874 specific proteins in the leaves of both varieties, including 1,965 differentially expressed proteins (representing 28.59% of all identified proteins, Table S1–S3). An analysis of the log_2_ fold-changes of the DAPs showed that, under PEG-induced water stress, the fold changes of DAPs were significantly higher in Y87 plants in relation to Y6 plants (Fig. S5, S6). A Venn diagram was constructed to illustrate the commonalities and differences of DAPs in Y6 and Y87 plants (Fig. 5A). After PEG-induced water stress, 228 proteins showed increased expression levels and 177 proteins showed decreased expression levels in Y6 plants, whereas 704 proteins showed higher expression levels and 856 showed lower expression levels in Y87 plants. Among these proteins, 114 proteins (consisting of 47 up- and 67 downregulated proteins) were common to both Y6 and Y87 plants (Fig. 5A). The gene ontology (GO) annotation analysis showed that these differentially expressed proteins (DAPs) were divided into eight biological processes, six molecular functions, and six cellular component subgroups (Table S4, S5; Fig. 5B,C). In Y6 plants, the pathways rich in DAPs were mainly related to oxidoreductase activity, followed by antioxidant activity, homeostatic process, and thylakoid functions (Fig. 5B). However, the four most significant DAP pathways in Y87 plants were protein-containing complex, organonitrogen compound biosynthetic process, cellular nitrogen compound biosynthetic process, and amide biosynthetic process (Fig. 5C). The enriched pathways of DAPs were further characterized by KEGG pathway analysis. The most markedly enriched DAPs in Y6 plants were related to photosynthesis, purine metabolism, and phenylpropanoid biosynthesis pathways (Fig. 6A). Contrarily, the pathways implicated with the largest percentage of DAPs in Y87 plants were ribosome, glycolysis/gluconeogenesis, oxidative phosphorylation, and photosynthesis (Fig. 6B).

### Identification of protein coexpression modules

**Figure 7:**
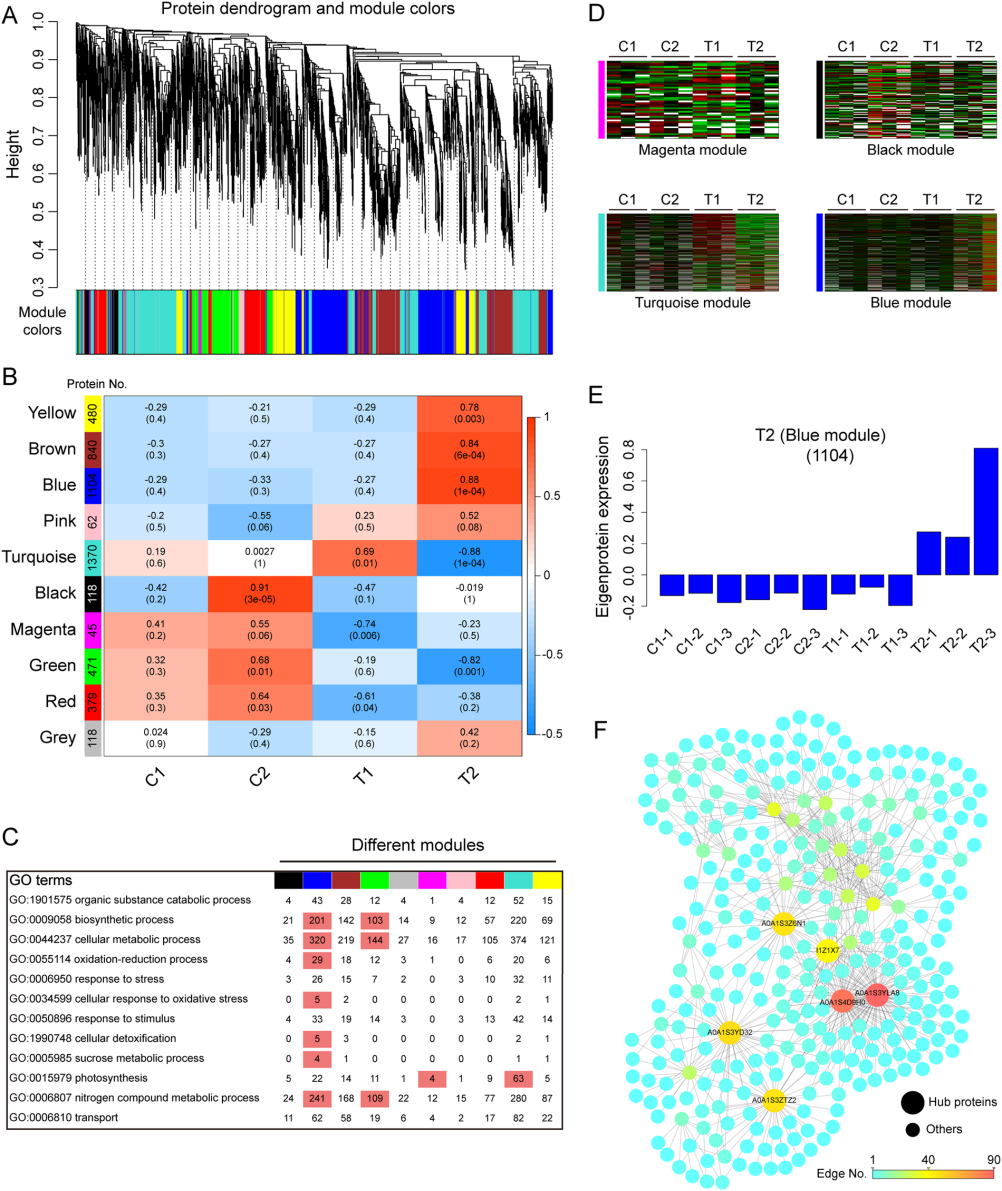
Coexpression network analysis with WGCNA. (**A**) Hierarchical clustering tree (dendrogram) of proteins based on coexpression network analysis. Each tree branch constitutes a module and each leaf in the tree corresponds to individual protein. (**B**) Module-trait relationship. The total number of proteins in each module is shown on the left. The color of each cell at the row-column intersection represents the correlation coefficient between the module and the sample. A high degree of correlation between a specific module and the treatment group is shown by dark red. (**C**) Ten modules were GO enrichment analysis by Fisher’s exact test. The number of proteins for each GO term of the corresponding modules is given. (**D**) Heat map showing the expression profile of the coexpressed proteins from four specific modules. (**E**) Eigenprotein expression profile for the blue (T2) module in different samples. The y axis indicates the value of the module eigenprotein; the x axis indicates sample type. The number of proteins in the module is indicated in parenthesis. (**F**) The correlation network of the blue (T2) module with the edge weight higher than 0.6. Six proteins with the edge number higher than 40 are indicated by larger circles among the 310 proteins shown in the network.

To investigate the protein regulatory network, we identified coexpressed protein sets in both tobacco plants via weighted gene coexpression network analysis (WGCNA). This network analysis resulted in ten coexpression modules (comprised of 45–1370 proteins) shown by the protein dendrogram (Fig. 7A, Table S6). Further, we associated each of the coexpression modules with four sampled tissues via Pearson correlation coefficient analysis. Notably, one coexpression module of Y6 and six modules of Y87 showed relatively higher correlation (r ≥ 0.60) with treatment groups (Fig. 7B). Many of these 10 modules were correlated with more than one treatment group; however, a few of them identify a specific treatment group only. For example, the black module was specifically correlated with C2 (0.91), and the turquoise and blue modules were specifically correlated with T1 (0.69) and T2 (0.88), respectively (Fig. 7B). GO enrichment analysis indicated different characteristics for the ten coexpression modules (Fig. 7C). A heat map showing the expression profile of each protein from four specific modules revealed that many of the turquoise module proteins are weakly expressed in T2 (Fig. 7D). Figure 7E shows the eigenprotein expression for the blue (T2) module (Fig. 7E). Proteins in the blue module were up-regulated after water stress and are highly correlated with alpha-aminoadipic semialdehyde synthase, glycosyltransferase, glyoxylate/succinic semialdehyde reductase, and aspartic proteinase, which appear to hub proteins that may regulate tobacco defenses to water stress (Fig. 7F).

### Quantitative PCR validation of iTRAQ data

**Figure 8:**
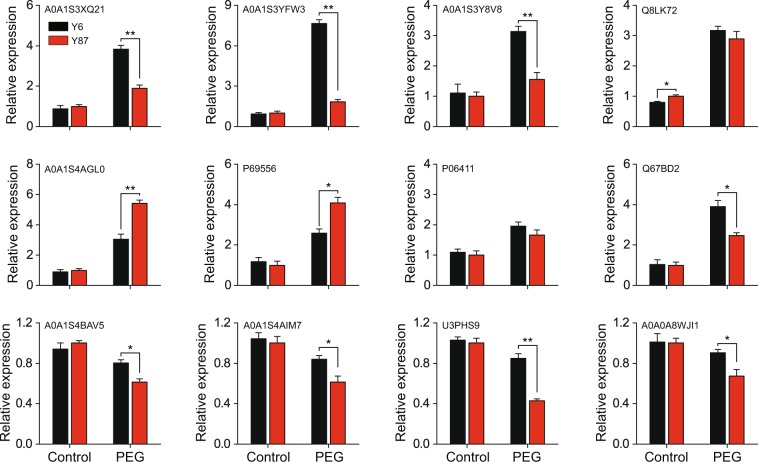
mRNA expression of twelve genes in Y6 and Y87 varieties after PEG-induced water stress. The expression level in Y87 under normal condition (control) was set to 1.0. Values represent mean ± SE of three replicated experiments. Asterisks indicate significant differences between Y6 and Y87 seedlings (Student’s *t*-test; *p < 0.05, **p < 0.01).

To investigate the similarity between protein levels and mRNA transcription patterns, we randomly selected twelve proteins from both varieties and monitored their mRNA levels using qPCR analysis. The fold change in transcript abundance of eight genes was similar to the changes in their protein levels, as indicated by the iTRAQ analysis, and only four genes showed poor correlation between mRNA and protein expression (Fig. 8), indicating that protein expression levels and transcript levels were similar for most of the selected proteins.

## Discussion

### Physiological response

Sen *et al*.^35^ reported that the enhanced drought resistance of transgenic tobacco plants is attributed to their increased sensitivity to ABA. Similarly, we observed an increase in the expression of the ABA biosynthetic gene, *NCED1*, in both tobacco varieties, under PEG-induced water stress, suggesting that ABA-mediated stomatal closure is associated with an increase in drought tolerance. Additionally, an analysis of the stomatal density also demonstrated that Y6 showed a higher water retention capability (given its stomatal density was lower than in Y87) and a higher water use efficiency (due to a decrease in water vapor diffusion). In agreement with previous findings^36,37^, our data suggested that non-stomatal factors, such as drought-induced oxidative damage, were responsible for inhibiting photosynthesis in Y87 plants under drought conditions. However, under the same degree of stress, photosynthetic inhibition in the drought-tolerant Y6 variety was mainly associated with stomata diffusion limitations, leading to a reduced carbon dioxide fixation efficiency. In addition to stomatal regulation, the action of the antioxidant defense machinery plays crucial roles in protecting plants from oxidative stress caused by drought. In this context, the balance between oxidant accumulation and removal by the antioxidant system establishes the intensity of oxidative stress. In the present study, Y6 and Y87 plants showed similar adaptive strategies to cope with oxidative stress associated to drought conditions. However, the drought-tolerant variety (Y6) exhibited higher tolerance to ROS-induced oxidative stress by activating its antioxidant systems more effectively, which may have contributed to the maintenance of cell membrane stability and photosynthetic activity in Y6 plants. In contrast, Y87 plants showed increased membrane lipid peroxidation, greater ROS accumulation, lower levels of antioxidants, and increased cell death after PEG-induced water stress. This suggests there are obvious differences in drought responses between Y6 and Y87 cultivars.

### Signal transduction

The 14-3-3 protein is one of the key signal transduction regulators. It regulates a variety of biochemical processes, including hormone cross-talk, transcriptional activation, and response to stress by interacting with target proteins^38,39^. It has been shown that overexpression of the *Glycine soja* 14-3-3 gene, *GsGF14o*, confers enhanced drought resistance in transgenic *Arabidopsis* by regulating stomatal size and root growth^40^. In the current study, two 14-3-3 proteins, Q75ZE5 and A0A1S4CMF0, showed upregulated expression in Y87 plants exposed to PEG-induced water stress, whereas no changes in the expression levels of these proteins were observed in Y6 plants, suggesting they play active roles in water stress. RNA-binding proteins (RBPs) have been considered crucial regulatory factors of RNA metabolism in diverse cellular processes. In particular, several RBPs have been found to play critical roles in abiotic stress responses by acting as RNA chaperones^41,42^. Our results indicate that the levels of the RBP 8A-like protein, A0A1S4A2P2, were dramatically increased in PEG-treated Y87 plants, similar to the proteomic response of maize plants subjected to water stress^9^. The upregulation of this protein suggests that it may act as a positive signaling molecule in the drought response signal transduction pathway.

### Carbohydrate and energy metabolism

In this study, we also observed that water stress modified the accumulation of proteins involved in carbon and energy metabolism. Compared to Y6, Y87 plants showed higher expression levels of ATP-dependent 6-phosphofructokinase, NAD(P)H-quinone oxidoreductase subunit M, and alcohol dehydrogenase aldehyde dehydrogenase (ALDH) family proteins after PEG-induced water stress. Increased accumulation of these proteins, which are related to the glycolysis and gluconeogenesis pathways, contributed to the enhanced defense system against water stress, similar to the proteomic data of a previous study^43^. The upregulation of ATP synthase, an important protein in maintaining energy homeostasis under stress conditions, alleviates drought stress in plants by markedly increasing ATP production to meet increased energy demands^44^. Our proteomics results showed that the ATP synthase subunit alpha and subunit beta were upregulated in PEG-treated Y87 plants, but were not affected in Y6 plants, indicating that the drought-sensitive cultivar (Y87) had a higher energy requirement to maintain cellular homeostasis during drought compared to the drought-tolerant cultivar (Y6). Conversely, we observed lower levels of two key proteins of the pentose phosphate pathway, transketolase and 6-phosphogluconate dehydrogenase, in stressed Y87 plants. The downregulation of these two enzymes may partially explain the growth inhibition of Y87 plants under conditions of water deficiency. The differential expression patterns of these proteins, which are related to carbohydrate and energy metabolism, in tobacco plants indicate that water deficit leads to changes in metabolic processes as a response to drought stress.

### Photosynthesis

The oxygen-evolving enhancer (OEE) protein is recognized as a key protein of the photosynthetic light reaction, and plays an important role in controlling O_2_ evolution and maintaining PS II stability^45^. In this study, we observed three upregulated OEE proteins in Y6 plants and six downregulated OEE proteins in Y87 plants subjected to PEG-induced water stress. This implies that the drought-tolerant variety (Y6) had higher a light-capturing ability compared to the drought-sensitive variety (Y87) during water stress. Rubisco is an enzyme that plays an important role in photosynthetic carbon assimilation and catalyzes the conversion of ribulose-1,5-bisphosphate into 3-phospho-glycerate^46^. We found that several rubisco proteins (A0A1S4D7A4, A0A1S4DIY1, A0A1S3Y035, and A0A1S4AKW3) were downregulated in both varieties after PEG-induced water stress. This indicates a reduction in the carboxylation rate in both tobacco varieties under drought conditions. Consistent with these results, the levels of chlorophyll a-b binding proteins were also dramatically decreased in Y87 plants treated with PEG. Contrarily, no obvious differences were found in the levels of chlorophyll a-b binding proteins in Y6 plants under drought conditions. These results imply that Y6 plants showed higher photosynthetic activity than Y87 plants, which may account for the significantly decreased net photosynthetic rate in Y87 plants that were exposed to water stress.

### Antioxidant defense

According to our iTRAQ data, four SOD proteins (A0A1S3XQ21, A0A1S4D5J3, A0A1S3ZTX1, and A0A1S4DS24) were significantly upregulated in Y6 plants exposed to water stress, while one SOD protein (A0A1S4DS24) was upregulated and another (A0A1S4D5J3) was downregulated in Y87 plants. In addition, 13 PODs were identified by iTRAQ, including five that were dramatically upregulated in PEG-treated Y6 plants and five that were upregulated in PEG-treated Y87 plants. The upregulation of these proteins, which was more pronounced in Y6 plants than in Y87 plants, suggest that PODs may protect plant cells from oxygen toxicity by detoxifying ROS. Consistent with these findings, SOD and POD enzyme activities were higher in Y6 plants than in Y87 plants subjected to water stress. Another ROS-related enzyme, glutathione S-transferase (GST), is of vital importance for plant cellular metabolism, detoxification, and defense against stress^43^. Patterns of GST accumulation implies that its expression (A0A1S3XWK1) was upregulated in Y6 plants after PEG treatment, but was significantly downregulated in stressed Y87 plants. Thus, the upregulation of this protein may be helpful to remove ROS in drought conditions. Delta-1-pyrroline-5-carboxylate synthase (P5CS), a key enzyme participating in proline accumulation, acts as a membrane structure stabilizer by regulating osmoticum under adverse conditions^47^. However, we found that, after PEG-induced water stress, levels of P5CS protein increased by more than 1.3 fold in Y87 plants but were unaffected in Y6 plants. These results indicate that the mechanism responsible for ROS scavenging differs in Y6 and Y87 varieties.

## Conclusions

**Figure 9:**
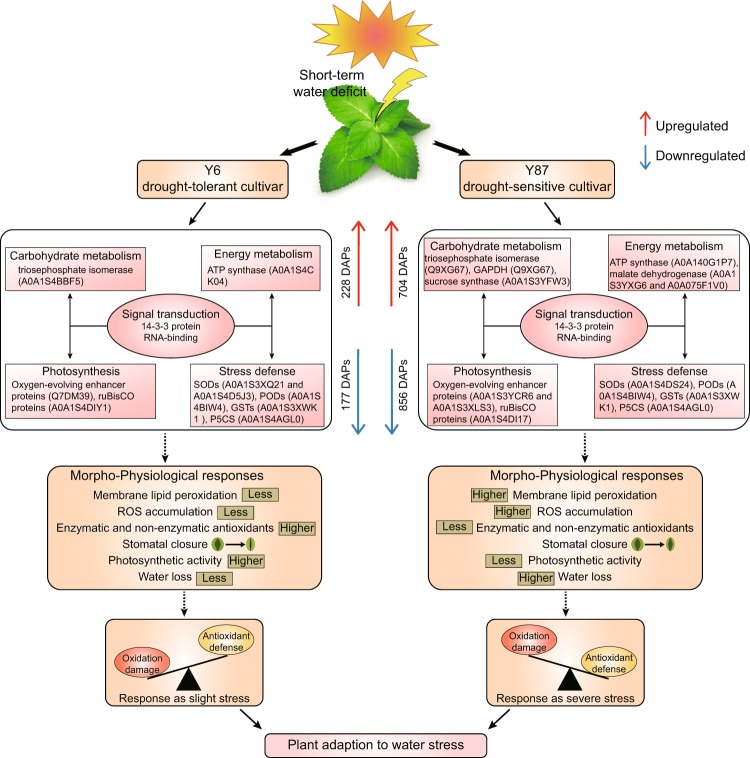
Schematic representation showing the differential responses of Y6 and Y87 varieties to water stress. Upregulated differentially expressed proteins are indicated by red arrows, whereas downregulated differentially expressed proteins are indicated by blue arrows. The drought-resistant Y6 variety exhibited better protein stability and physiological status in comparison to the drought-sensitive Y87 variety during water stress.

In summary, comparative analyses of morphology, physiology, and proteomics provide comprehensive insights into the overall and variety-specific mechanisms underlying drought response in two different tobacco cultivars. The differential accumulation of several proteins involved in signal transduction, carbohydrate metabolism, energy metabolism, photosynthesis, redox homeostasis, among other biological processes, is vital for tobacco plants to respond to drought stress. There were 704 upregulated and 856 downregulated DAPs in drought-sensitive Y87 plants, but only 228 and 177, respectively, in drought-tolerant Y6 plants, indicating that Y6 plants exhibit a greater ability to maintain protein stability in relation to Y87 plants (Fig. 9). These proteins act cooperatively to provide metabolic homeostasis enabling plant response against water stress, consistent with the physiological performance observed in both varieties. However, we believe further work is required to verify the role of these novel drought tolerance-related proteins using transgenic experiments.

## Materials and Methods

### Plant materials, growing conditions, and stress treatments

Flue-cured tobacco varieties, Yuyan6 (Y6) and Yunyan87 (Y87), were used in this study. Tobacco seeds were provided by Henan Agricultural University (Zhengzhou, China). Seeds were sterilized for five minutes in 10% sodium hypochlorite and germinated in an illuminated incubator. Fourteen days after germination, tobacco seedlings were transplanted into pots containing pre-sterilized perlite (four plants in each pot). Subsequently, these were moved to a growth chamber with controlled environmental conditions: 16 h light/8 h dark cycle, temperatures of 25 °C/18 °C (day/night), light intensity of 200 μmol m^−2^ s^−1^, and relative air humidity of approximately 60%. Seedlings were watered with half-strength Hoagland nutrient solution every day.

To determine the effects of drought stress on seed germination, aseptic seeds from Y6 and Y87 plants were put in Petri dishes containing a 15% (w/v) PEG6000 solution and incubated at 25 °C with a 16-hour photoperiod. Germination rates were recorded daily and seed germination phenotype was examined seven days after germination. For soil drought treatment, 5-week-old plants were transferred into plastic pots (9 × 14 × 13 cm) containing a mixture of peat and vermiculite (2:1, v/v). After three weeks in the glasshouse, the tobacco plants were not irrigated for 16 days until a severe drought effect was observed. At that moment, soil moisture content was gradually reduced to approximately 15%. Soil moisture was measured daily using a Soil Moisture Meter (Tuopu Bio Co., China) as described by Xia *et al*.^48^. Control seedlings were watered regularly. Leaves from both control and water deprived plants were used to evaluate the maximal efficiency of PSII photochemistry (Fv/Fm) and chlorophyll content.

For the PEG-induced water stress treatment, 5-week-old tobacco seedlings grown in pots were shifted to containers containing Hoagland’s solution and allowed to grow for another seven days. Osmotic stress was then applied to plants with a consistent growth state by adding a 15% (w/v) PEG6000 solution. The osmotic potential for 15% PEG solution has been previously estimated at −0.73 MPa^49^. For the ABA treatment, plant leaves were sprayed with 100 µM ABA solution. All samples were collected at the indicated time points and stored at −80 °C for subsequent analyses. Leaf tissues were sampled from the youngest fully developed leaf.

### Observation of leaf ultrastructure

Stomatal aperture and stomatal density were detected using an SU8010 scanning electron microscope (Hitachi, Tokyo, Japan). Additionally, chloroplast ultrastructure of leaves was visualized using an H-7650 transmission electron microscope (Hitachi, Tokyo, Japan).

### *In vitro* leaf senescence assay

Leaf discs punched from completely expanded leaves of 6-week-old seedlings were used to assess leaf senescence. The leaf discs were put to float on a nutrient solution supplemented with 15% PEG for eight days under light. The anatomical structure of the leaf discs was observed with safranin O/fast green staining based on Wei *et al*.^50^. The chlorophyll content of the leaf discs was measured at 2, 4, 6, and 8 days using a SPAD-502 Chlorophyll Meter (Minolta Camera Co. Ltd., Japan).

### Determination of Photosynthetic efficiency

The Fv/Fm ratio was measured using a PAM-2100 Chl fluorometer (Walz, Germany). An LI-6400 Portable Photosynthesis Analyzer (LI-COR, USA) was used to measure net photosynthesis (Pn) and stomatal conductance (Gs) as described by Huo *et al*.^51^.

### Measurement of physiological parameters and histochemical observations

The physiological parameters were analyzed using the 3rd youngest leaves of three plants/treatment in each replicate. Measurements of leaf RWC and chlorophyll content were performed as described by Hu *et al*.^52^. EL was determined according to Dahro *et al*.^53^. MDA content, ROS (O_2_^•−^ and H_2_O_2_) levels, and the activities of anti-oxidative enzymes (SOD [E.C. 1.15.1.1], POD [E.C. 1.11.1.7], CAT [E.C. 1.11.1.6], and APX [E.C. 1.11.1.11]) were examined using specific detection kits (Jiancheng Bioengineering Institute, Nanjing, China), according to the manufacturer’s instructions. The accumulation of osmoprotectants (proline and soluble sugars) was examined as described by Zhao *et al*.^54^. AsA and DHA levels were measured according to Xing *et al*.^55^. *In situ* accumulation of O_2_^•−^ and H_2_O_2_ were detected by histochemical staining with nitroblue tetrazolium and diaminobenzidine, respectively^35^. Starch content was histochemically detected using potassium iodide staining, as described by Yang *et al*.^56^. Intracellular ROS production was detected by dihydroethidium staining and then visualized under a fluorescence microscope (Eclipse TI-SR; Nikon, Tokyo, Japan). A TUNEL apoptosis assay was used to assess PCD based on Li *et al*.^57^. Three independent replicates were performed for every experiment.

### Identification and functional analysis of DAPs

**Figure 10:**
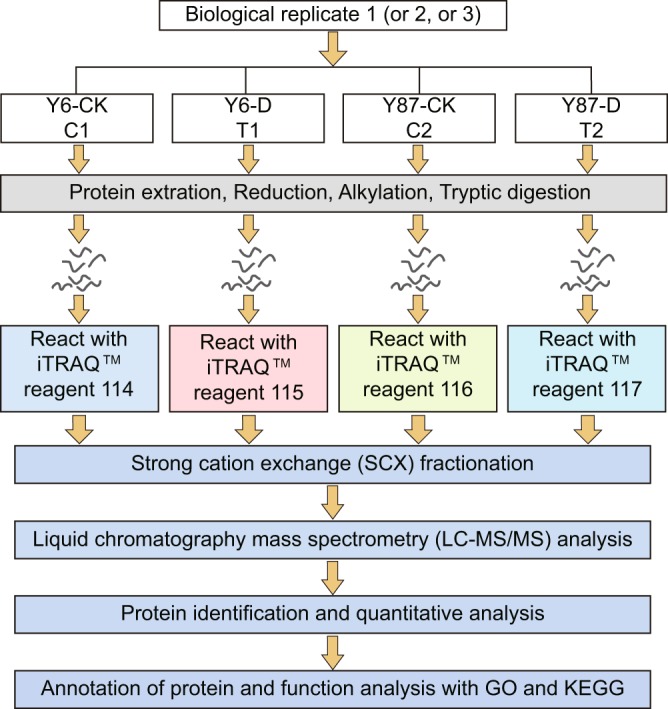
Schematic representation of iTRAQ 4-plex labeling used to identify drought-resistant proteins in seedling leaves of both tobacco varieties under control (CK) and water stress (D) conditions.ss

After two days of PEG-induced water stress, proteins were extracted from leaf samples of both varieties and digested as previously described by Long *et al*.^58^. Peptides from the twelve extracted samples were labeled with three sets of 4-plex iTRAQ reagents (Applied Biosystems, Foster City, CA, USA), following the manufacturer’s instructions (Fig. 10). In the present study, non-stressed Y6 (C1), stressed Y6 (T1), non-stressed Y87 (C2), and stressed Y87 (T2) were labeled with the iTRAQ tags 114, 115, 116, and 117, respectively. Labeled peptides were then analyzed by strong cation exchange and liquid chromatography-tandem mass spectrometry according to Zhang *et al*.^59^. Identified proteins that differed between stressed and non-stressed plants with a fold change >1.20 or <0.83 (*p* < 0.05) were defined as significant DAPs. Functional characterization and metabolic pathway enrichment analyses of DAPs were performed based on GO terms and the KEGG database^60,61^, respectively.

### Quantitative PCR

Transcriptional expression profiles of target genes were determined using a CFX 96 Real-Time PCR Detection System (Bio-Rad, CA, USA) with the specific primers listed in Table S7. The tobacco actin gene was selected as an endogenous control and the expression pattern of each gene was analyzed according to the 2^−ΔΔCT^ method^62^. Each sample was examined three times and each independent biological experiment contains three technological replicates.

### Statistical analysis

The physiological assay and qPCR results were analyzed using SPSS 23.0 software (IBM Analytics, NY, USA) and significant differences between Y6 and Y87 plants were assessed using the Student’s *t*-test.

## Supplementary information

Supplementary Information

Supplementary Information: Table S1

Supplementary Information: Table S2

Supplementary Information: Table S3

Supplementary Information: Table S4

Supplementary Information: Table S5

Supplementary Information: Table S6

Supplementary Information: Table S7

## Data Availability

All mass spectrometry data was deposited into the publicly accessible database iProX (http://www.iprox.org/) with identifier IPX0001706000.
