# Supplementary material for: Morpho-physiological and proteomic responses to water stress in two contrasting tobacco varieties
Source: Sci Rep. 2019 Dec 6;9:18523. doi: 10.1038/s41598-019-54995-1 (PMC6898209; doi:10.1038/s41598-019-54995-1)
Supplement: Supplementary file 1 — Supplementary Information [file 41598_2019_54995_MOESM1_ESM.pdf]

Morpho-physiological and proteomic responses to dehydration stress in two contrasting tobacco varieties

Zheng Chen[1], Jiayang Xu[2], Fazhan Wang[1], Lin Wang[1] & Zicheng Xu[1,*]

[1] College of Tobacco Science, Henan Agricultural University, Zhengzhou 450002, China

[2] Agronomy and Biotechnology College, China Agricultural University, Beijing 100193, China

[*] Corresponding author.

Prof. Zicheng Xu

College of tobacco science, Henan Agricultural University, Zhengzhou 450002, China.

Email: zichengxu@126.com

Supplementary Figures

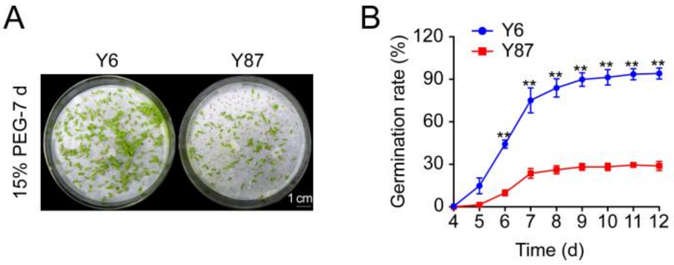

Supplementary Figure S1. Seed germination phenotype (**A**) and time-course germination rates (**B**) of Y6 and Y87 on filter paper containing 15% PEG solution (n = 350).

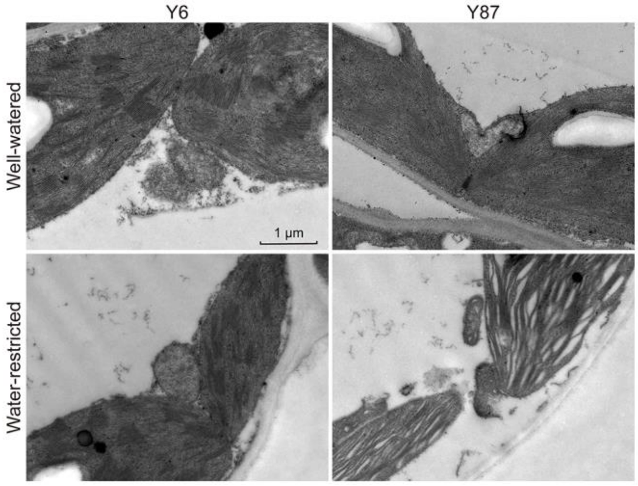

Supplementary Figure S2. Transmission electron microscopic photos of chloroplasts from Y6 and Y87 seedling leaves under normal and PEG stress conditions.

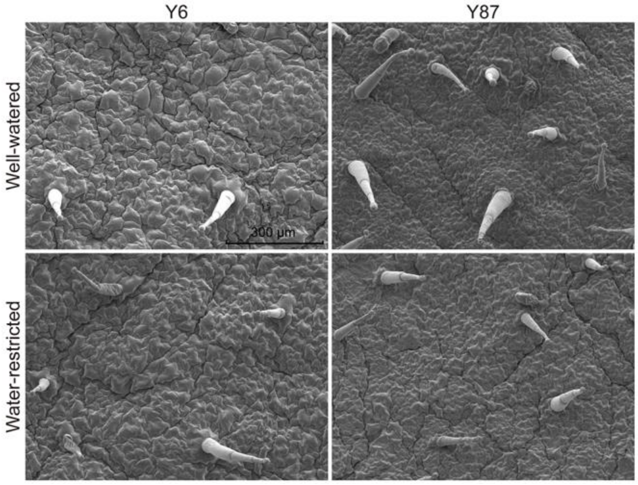

Supplementary Figure S3. Stomatal density of leaf abaxial epidermis in Y6 and Y87 seedling under normal and PEG stress conditions via scanning electron microscopy observation.

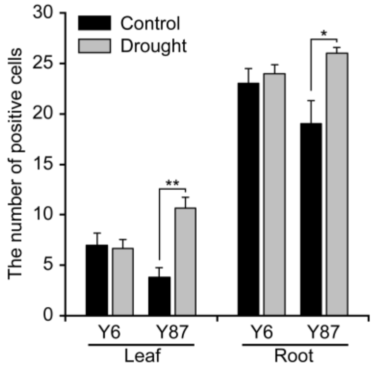

Supplementary Figure S4. Quantitative analyzed the PCD cell in well-watered and PEG-stressed tobacco plants. Bars represent ± SE calculated from three replication experiments. *$P < 0.05$ and **$P < 0.01$ denote significant differences between control and treatment groups from the same tobacco varieties.

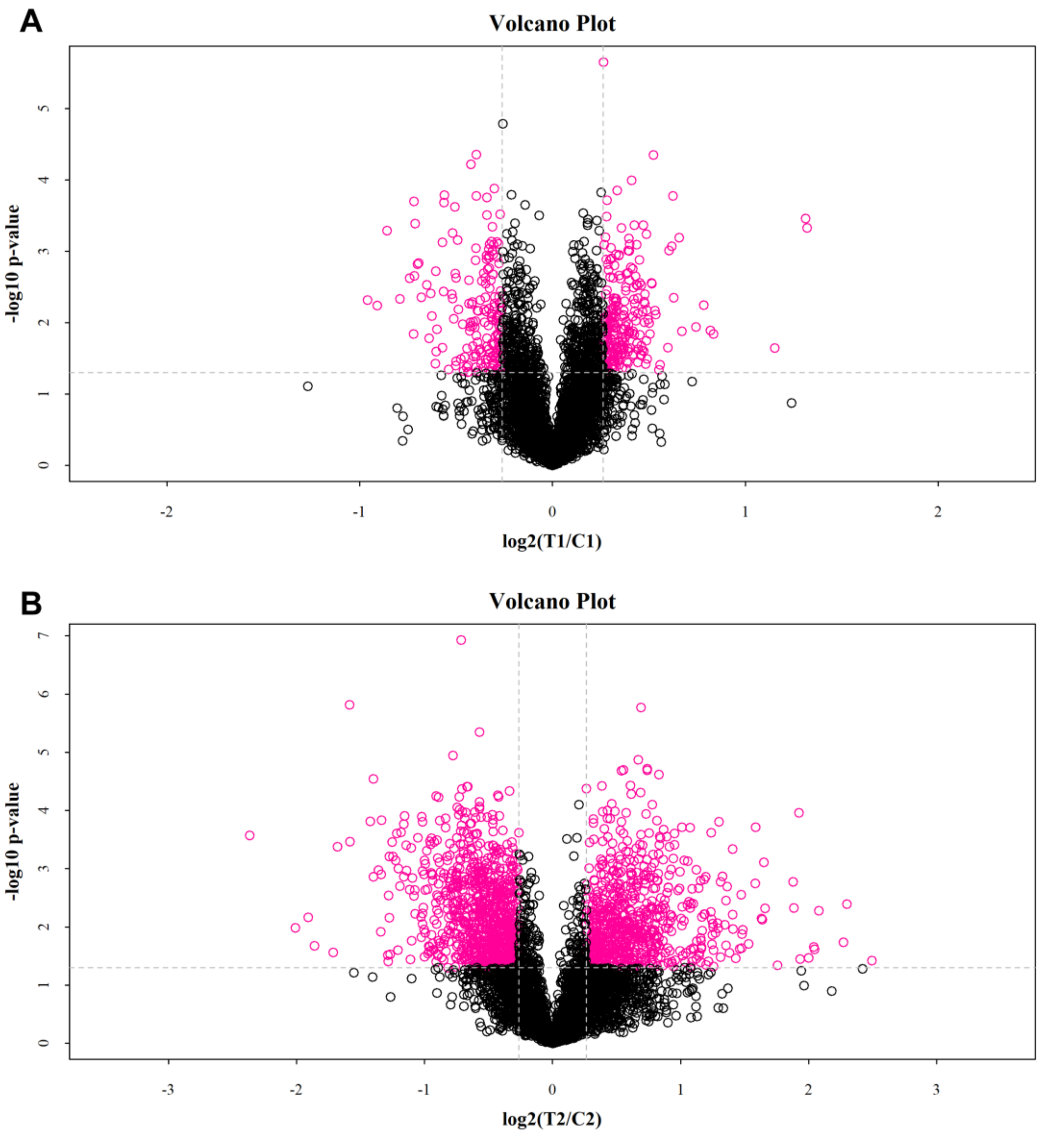

Supplementary Figure S5. Volcano plot showing quantitative and differential analysis of DAPs in drought-tolerant Y6 (**A**), and drought-sensitive Y87 (**B**) under PEG stress. The horizontal axis shows the log2 fold change expression; the vertical axis shows the significant different *p*-value (log10 transformation). The red dots are significant differentially abundant proteins (multiples vary more than 1.2 times and *P* value < 0.05), and black dots are no differential changes in the proteins.

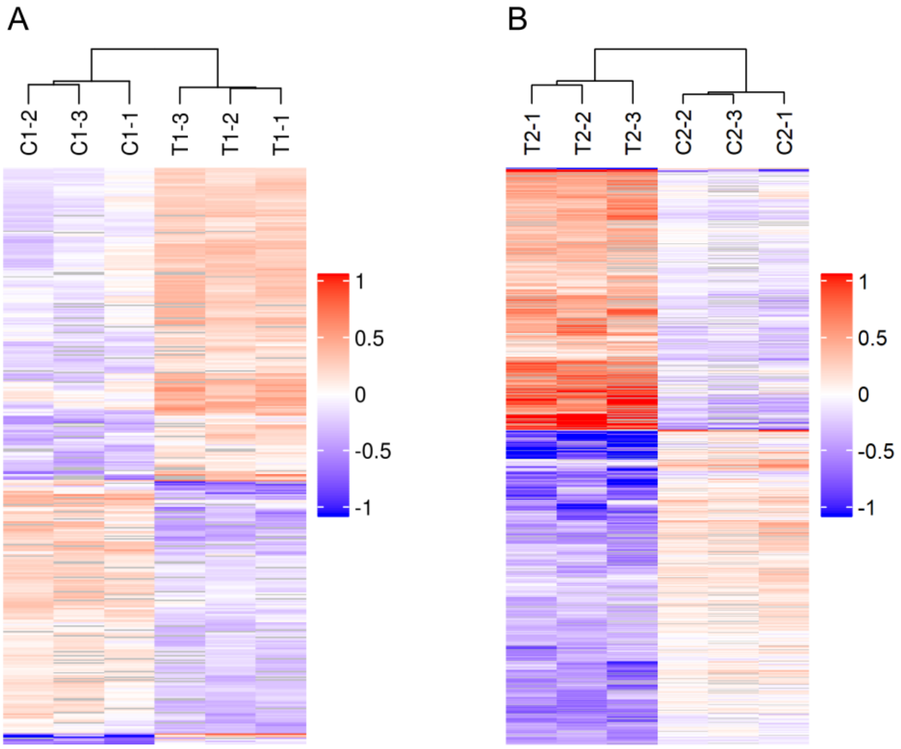

Supplementary Figure S6. Clustering analysis of significant DAPs in (**A**) Y6 before and after PEG treatment (T1/C1); (**B**) Y87 before and after PEG treatment (T2/C2). Each row represents a protein significantly abundantly expressed. Columns represent sample replicates. C1 and C2 showing sample replicates under well-watered conditions for Y6 and Y87, respectively; T1 and T2 for Y6 and Y87, respectively; The scale bar on the X-axis indicates the logarithmic value (log2 expression) of the expression of significant DAPs in different samples, up-regulated (red) and down-regulated (blue). Basically, more DAPs showed higher expression (and up-regulation) in Y87 than Y6.

Supplementary Tables

Supplementary Table S1. List of identified proteins in Y6 and Y87 varieties.

Supplementary Table S2. Differentially accumulated proteins (DAPs) in the leaves of Y6 plants subjected to PEG-induced dehydration stress (T1/C1).

Supplementary Table S3. Differentially accumulated proteins (DAPs) in the leaves of Y87 plants subjected to PEG-induced dehydration stress (T2/C2).

Supplementary Table S4. Enriched GO terms of the DAPs in Y6 plants subjected to PEG-induced dehydration stress (T1/C1).

Supplementary Table S5. Enriched GO terms of the DAPs in Y87 plants subjected to PEG-induced dehydration stress (T2/C2).

Supplementary Table S6. Results of WGCNA analysis.

Supplementary Table S7. Primer sequences used for qPCR analyses.
